# The Importance of Intelligent Nursing and the Training of Nurses

**Published:** 1885-07

**Authors:** Howard J. Williams

**Affiliations:** Orator, Macon, Georgia


VOL. II. JULY, 1885. NO. 5.

### Original Communications.

### THE IMPORTANCE OF INTELLIGENT NURSING AND THE TRAINING OF NURSES.

AN ADDRESS DELIVERED BEFORE THE MEDICAL ASSOCIATION OF GEORGIA, AT SAVANNAH, APRIL 15TH, 1885, BY HOWARD J. WILLIAMS, M. D., ORATOR, MACON, GEORGIA.

*Mr. President and Gentlemen:*

Another year, filled with its joys and success to some, its sorrows and conflicts to others, has passed, and again we are gathered together for a season of rest and relaxation, while we discuss measures concerning our profession and the health and happiness of our race.

Looking around me, I am struck with the thought, if I could but see into the soul of each one present this evening and read the experiences of the past year, I would find a strange interweaving of the sweets and bitters, the sunshine and the clouds of life. I trust, however, that the sweets and sunshine have predominated and that all clouds were only transitory. The world is cold and bitter to some, and it seems that our profession is too often made to bear the bitter complaints, while the smiles are saved for others. Yet, sometimes we are treated to a little sunshine, and occasionally we receive a few sweets, in order that we may better relish the bitter. Perhaps some patient, appreciating your efforts in his behalf, has sweetly urged, if you have not prescribed for all his ailments, that you are careless and indifferent; and, when to please him, you did prescribe, he kindly said, "you deluge me with physic." The admiring public thought because your horse was fat you had nothing to do, and gently murmured when he was lean, "you were too infernally stingy to feed him." When your patients recovered you were told it was all due to the good nursing they received; if they died, you were made to understand that you did not understand their cases. When you did not go as soon as sent for, you were accused of taking things too easy; but when you sent in your bill, you were informed that "you were in a terrible hurry for your money and you must wait awhile." Visiting your patient every day was only to run up a bill; failing to call was an unjustifiable neglect; and, if you have received pay for one-half of the last twelve months' services you are very fortunate, and deserve to be immortalized. Such, then, are a few of the sweets of a physician's life, and he who does not appreciate them is without a soul, and unfit for such a world of sunshine and happiness.

But to turn to the duties of the hour, I must express my deep appreciation of the honor conferred upon me of addressing you to-night, yet I am painfully conscious of my unworthiness of the position and my inability to fill it with credit to myself and pleasure to you. I am no orator and cannot inspire your souls with lofty flights of eloquence, nor am I an investigator to add to your store of medical knowledge new and striking discoveries in science. If, however, I can impress upon your minds something, the importance of which we all recognize, and without which our efforts at overcoming disease will be unsatisfactory, I will feel that I have not altogether failed in performing my task.

No new discoveries in medicine, or grand triumphs of surgery, will remove disease from the human race, nor place man beyond the need of watchful care while struggling for life and health. Since this struggle must continue from infancy to old age, and without watchful care it would soon end in death, the duty of caring for the sick demands our most earnest consideration, and I have, therefore, selected for my subject,

"THE IMPORTANCE OF INTELLIGENT NURSING AND THE TRAINING OF NURSES."

When the functions of health have been interfered with and disease is produced, nature endeavors to remove the interference and repair the damage. The success or failure of her efforts will depend largely, in fact, mainly, upon her surroundings. If the victim of disease is denied rest, both of mind and body, the air he breathes be impure, his nourishment insufficient and improper, himself, his bedding and his surroundings filthy, nature has but poor chance of carrying out her work of repair. Unassisted, she will fail; but surround her with conditions favorable to health and her success is assured. Intelligent nursing will assist nature by providing her with these conditions, thus being her hand-maid.

It is claimed that medicine is curative. Not so; medicine is no more curative than surgery. The surgeon removes the bullet, but nature heals the wound. The obstacle is removed, the parts are placed in a condition favorable to repair, but nature's own hand and skill can alone fill up the gap and restore beauty and comeliness to the parts. Medicine is but the surgery of functions; it removes obstacles, but nature restores the functions to healthy action. Medicine is the science of curing disease and preserving health; nursing is the art of caring for the sick and aiding nature to restore life and strength. Medicine and nursing aid each other—the one prescribes, the other applies the remedies. The former is useless without the latter.

The art of nursing is all but unknown, and its value comparatively unrecognized. The popular idea of nursing embraces the mere administration of physic, the application of poultices, and keeping the patient in bed and the bed clothes on the patient. Intelligent nursing embraces more than this—it recognizes that the laws of health are as necessary for the sick as for the well, and endeavors to enforce them in the sick-room. It supplies the sick with rest, air, warmth, light, cleanliness and food as they are needed, and at the least expense of vital force. It relieves the sick from all thought of self and prevents all unnecessary exertion. Standing guard over the sick, it perceives the unexpected changes in the disease, and by ready thought and quick action it arrests the approach of death and preserves life. All is eagerness for nature's service, and the study of her aid is never tiresome.

One of the most important of nature's aids, and one which intelligent nursing will seek to supply, is rest. Rest was man's first remedial agent, the sole article of his *materia medica*, implanted in him by a merciful providence to heal the wounds and soothe the pains that attend his accursed lot. And man has ever, when stricken with pain or overcome by disease, turned to this panacea, and yielding to its gentle influence gradually regained health and ease. In infancy and the growing years, rest is the principal agent in the development of tissue and the perfection of life, while in health it restores the weary powers and renews waste. If this be true of growth and health, how much more necessary of disease, when all is waste and destruction. The faithful nurse will therefore watch with jealous care her patient's hours of repose, nor allow one thing to disturb his rest. Every means of rest of body, every position will be studied as nature's index for aid and essential to her methods of cure. She well knows that in most diseases recumbency is assumed as if by instinct; that in others it is torture, and learns that other positions will then give ease. She recognizes the value of elevation as a means of rest to a congested, painful member, and she sees that exclusion of light is rest to an inflamed eye.

Rest of mind is as essential as rest of body. Mind and body are mysteriously united, and the influence of one over the other is inexplicable. Anger quickens the heart and flushes the face, and extreme fear has whitened the hair in a single night. In disease the mind is excited and the emotions are easily roused; the trained nurse knows, therefore, that all causes of excitement, great or small, should be avoided, for they disturb the mind and do incalculable harm. Careful attention to the minor details of the sick-room, and what might be called the petty management of the sick, will do much to prevent the mind from exerting an unwholesome influence over the body. Punctuality, conciseness, decision and calmness of the attendant have a healthy effect on the mind. Variety in the surroundings and occupation of the sick contributes to mental repose, and the mental effect of seeing the beautiful during illness must be experienced before it is truly appreciated. The intelligent nurse seizes upon these means of rest and turns them for good to the sufferer and help to nature.

For nature to successfully carry on her work of repair, it is necessary that the air of the sick-room should be fresh and pure. The demand for good ventilation is as imperative as the demand for rest. Good ventilation means the admission of a current of pure air into the sick-room, the airing of the bedding and clothes, the airing and disinfection of all the utensils used by the patient—in fact, the exclusion of all possible sources of infection in the air the patient breathes. This is but the fulfillment of one of nature's laws of health, for pure air is necessary to health. Foul air is the cause of infection, the bearer of germs, the disseminator of disease, and the only safeguard against its ravages is through ventilation and drainage. In nursing the sick, it is criminal to deny fresh air, for the air of the sick-room is loaded with the products of waste, thrown off by the exhalations from the lungs and skin. This air is poison, and with every breath the patient inhales he takes in that much poison. If ventilation is necessary in medical cases, it is yet more necessary in surgical and puerperal practice, for septicæmia and pyæmia are ever present in the air, loaded with the products of suppuration. Intelligent nursing recognizes the need of ventilation and the evils of foul air, and the faithful nurse will not neglect the one nor ignore the other.

Warmth is also necessary for nature's work of repair. The bodily temperature varies at different hours in the day, and it is especially apt to fall in the early morning. If the vital forces are much depressed, it is absolutely necessary to watch the temperature at this time, for patients are frequently lost in the latter stages of diseases from the neglect of this simple precaution.

The means of keeping up the animal warmth should be well understood by the nurse, and she should constantly be on the lookout for changes in temperature. Ventilation and warmth are equally necessary for successful nursing, and they should not interfere with each other; yet no question is more puzzling to an inexperienced nurse than how to ventilate, and at the same time keep the patient warm. If the patient is warmly clad in bed, and stimulants and hot applications are employed, ventilation by open windows can be accomplished.

Sunlight is also needed in the treatment of disease. For the growth of all vegetable and animal life sunlight is necessary. Plants raised in the dark are puny, and without sunshine children are dwarfed, scrofulous and rickety, and man degenerates body and mind. The sick man involuntarily turns his face to the window to see the light, seemingly impelled by nature to seek this medicine so bountifully given, and yet so frequently denied. Throw open the windows in abeyance to nature, and let pure air and sunshine come in to the sick man. These nature gave to man for life and health before man essayed to treat disease.

The next essential for good nursing is cleanliness, and few persons, even physicians, recognize its importance. Cleanliness and ventilation go hand in hand; the one is useless without the other. Often we are disgusted with the filth we encounter at the bedside, and yet we ignore the importance of prescribing cleanliness. What to the physician is but a trifle to be patiently endured is a cause of sickness, delayed recovery or death. Personal cleanliness is of prime importance. In almost all diseases the functions of the skin are disordered, and the skin is the channel for removing the products of waste. If the secretions of the skin are not removed they are reabsorbed—the poisoning by the skin is as certain as poisoning by the mouth. Who has not noticed the expression of relief and improvement depicted on the countenance of the sick after a bath and change of linen and bedding? Nor is personal cleanliness alone essential; the apartments, the walls, the furniture and all utensils must be clean, for they catch and retain organic matter floating in the air, and are sources of poison.

Of no less importance to the sick man than rest, air and cleanliness is food. Every secretion, every excretion, every function in life is attended with tissue change and loss. This loss must be restored by new tissue, which is obtained from the blood, and blood is food acted upon by digestion and assimilation. This being true in health, it is equally true in sickness, for not only is there waste from functional activity, but in addition a greater waste from disease. Food in sickness assists nature, in that it not only replaces the loss of tissue, but supports the system and increases the power of enduring disease. Nature can best be aided in this by regulating the patient's diet, giving such articles as are known to be strengthening, in the form in which they are assimilated with the greatest ease, and at times when they are most apt to be absorbed by the system. Yet no rules can be laid down for dieting the sick, and all attempts to formulate a dietary from analysis of food is but theory. Organic chemistry may teach us the amount of carbon needed to keep the body warm, or how much nitrogen is wasted in muscular action, but it cannot tell us anything about the reparative process going on in disease, nor indicate the diet for the sick. It is indeed not only what kind of food is given, but how it is given and when, that constitutes good nursing. Patients have often starved in the midst of plenty because the food was unpalatable or the delicate stomach could not retain it. Punctuality in giving food is often the secret of success in apparently hopeless cases, for life often depends upon the stimulating effects of food, and patients have often been lost by indifference or mistakes in regard to this matter. Careful nursing will assist the doctor by watching nature manifest herself in her choice of food for the sick.

In regard to the administration of medicine, a knowledge of the action of the principal drugs and their doses, the action of poisons and their antidotes, the preparation and application of local agents, and the use of certain instruments, is essential. The beneficial effects of drugs can often be ascertained in a short while, and if the nurse knows what to look for, she may be able to save time and life. Again, the condition of receptivity to some drugs varies at different times during an illness. An intelligent observer can recognize this and apply it with benefit to the sick. How many hours of suffering and broken rest could be saved if the nurse were instructed in the indications for, and use of, such instruments as the catheter and hypodermic syringe. Promptness and discretion in the administration of medicines and use of instruments are of great value when attended with a knowledge of their effects and uses. There is much to be learned in this department of nursing, apparently of minor import, yet so valuable that a nurse is inefficient who does not know them.

One thing of vast importance in nursing is intelligent observation. That it is required in practice every day can be readily appreciated when we see how little dependence can be placed in what is told us by the friends of the sick, for those unacquainted with disease cannot recognize its changes nor perceive the action of medicines. Observation is the basis of all good nursing, and much of diagnosis, prognosis and therapeutics depends upon its thoroughness. It is an art only to be attained by patience and untiring attention at the bedside, and years of faithful study is required to learn how and what to observe. A mere collection of varied information is not sufficient; there must be a reason for every fact observed, or observation is fruitless. Every disease, every modification of disease, manifests itself in some way by which an acute observer can detect the changes that are constantly occurring; and the object of observation is to detect these changes and ascertain nature's needs so that they may be intelligently supplied. Pathology, the science of morbid processes, is making rapid strides, yet bedside observation, equally as important, falls far short of the position it should occupy. We need a more intimate knowledge of the daily and hourly changes in disease, and a better understanding of the effects of our medicines and treatment. This can only be ascertained by constant observation, and intelligent nursing will supply us with the knowledge desired.

These and many other things are necessary for the success of nursing; and for the well-being of our patients, for the successful assistance of nature, and for our own efforts to subdue disease, it is essential to have associated with us those who are trained to care for the sick. Frequently we hear it said that such an one is a born nurse. Such a statement is a fallacy. No human being was ever endowed with such knowledge at birth, and the nurse without training is like the child with burnt fingers. Experience is gained only through blunders and misfortunes; but unfortunately it is the patient, and not the nurse, who is the victim of the blunders and suffers the misfortunes. Ignorance, mistaken kindness, or positive neglect, are frequent causes of death. Ignorance of the laws of health, fear of disturbing the sick, or indifference to the results, are all causes of neglect entirely beyond the control of the physician. Place at his disposal a person well trained in the art and guided by a feeling of responsibility, and disease is easy of control, and the chances of success are great.

Woman, by her nature, is best suited for the duties of a nurse; helpful and kind by instinct, generous and devoted by nature, her quiet influence in the sick-room is as a messenger of health and strength. She, following in the footsteps of her Master, would imitate Him in all things pure and noble, and a mission such as this is suited to her devoted spirit. There is no nobler mission, except the ministry, than that of nursing the sick. She who would choose it should be inspired alone by a generous devotion to the well-being of her race, a firm purpose to fulfill the object of life, and a noble sacrifice of self and all else for the sufferings of others. No romantic abandonment of self because of disappointed affections; no feelings of disgust at the supposed emptiness of the world; no feeling of incapacity for any other path in life, should be the motive for entering upon the sacred mission of a nurse. Popular writers of romance turn their broken-hearted heroines into devoted nurses, clothing them in a few hours with all the qualities acquired alone by years of experience and study. God pity any poor suffering being that falls into the hands of such mushroom nurses. Misanthropes, sneering at the world because of their own selfishnesss, are as little suited for the sick-room as the school-girl moping over her lost lover. But of all miserable impositions upon sick humanity is the nurse who, incapacitated for other walks in life, thinks in nursing she has found her mission.

The nurse must be chaste—so pure that an immodest jest could never be uttered in her presence; sober in spirit and temperate in all things; honest with her patient, her doctor and herself; truthful in all things, her words and actions above suspicion; punctual to the second and orderly to a hair; quiet and quick in her motions; cheerful and hopeful, and self-forgetful, thinking only of her patient and his wants. To these gifts, with which nature has endowed her, she must add knowledge derived from thorough training. Training teaches her to observe, to understand, to know and to perform exaetly everything necessary for life and health, and to guard against disease and death; and it teaches her in all things to fulfill her duty, not as a machine, but as an intelligent nurse; not servile, but loyal as to her conscience and her Master.

There are those who would urge that woman should know nothing of physic, for when they do they are continually physicking those around them. Though there may be some of whom this may be true, yet a true nurse will remember that it is the physician's part to prescribe and her duty to obey his directions. Women, as a general rule, are failures when they undertake to practice medicine. Occasionally one more brilliant than the rest succeeds, but she is the exception and not the rule. While the practice of physic is not woman's fort, she should be instructed in the science of hygiene and preventive medicine, for upon her we depend mostly for personal and household hygiene.

Since trained nurses are so much needed, and since women are best adapted to the business, it seems to be only our duty to train them for the purpose. To educate nurses in our towns and cities where there are no large hospitals, the physicians of the place might unite and form training schools, in which those who desire might be instructed in the art of nursing. In these schools the nurses could learn the principles of hygiene, the nature, preparation and administration of food, the effects, doses and modes of administering medicines, the action of poisons and their antidotes, how to apply surgical, obstetrical and gynecological dressings, the making and application of bandages, the management of the patient and the arrangement of the bed and sick-room. In every city and town there are a great number of sick among the poor who need just such care, and are desiring training in the art might learn the details among them under the guidance of a practitioner. By means of these schools we would be supplied with good, honest, faithful nurses, and our efforts to overcome disease would be successful.

I cannot close without expressing a few words of respect and veneration for one who has done so much to raise the art of nursing from a menial position, and given it its true place in science. Florence Nightingale, the child of cultivated and wealthy parents, gifted with no mean intellectual development, and surrounded by everything conducive to a life spent in fashionable society and æsthetic inactivity, chose rather to turn her attention to the study of sanitary reform, and devote her life to caring for the sick. From early childhood nursing the sick was to her a peculiar delight, and no reading so attractive as that pertaining to hospitals and institutions for the diseased and infirm. She early in life recognized that nursing was a science, and should be practiced only by those trained for it. She studied nursing as a science and reduced it to the practice of an art. Her services in the Crimea secured to her the sincerest gratitude of the British people and a world-wide renown. The soldiers, won by her gentle manner and kindly smile as she moved among them, regarded her with feelings akin to idolatry. So noble, so generous, so sublime in her devotion and untiring zeal, yet with all, so modest, she was a queen among nurses. No page of history reflects the light of a nobler life than that which records her devoted and self-sacrificing service to suffering humanity. A higher type of womanly character could nowhere be found, and one of which true womanhood might well be proud. They who waste their lives in idleness and selfish pleasure need never expect to gain anything of that higher and better life which devotion to duty has given to her. In shame let them hide their faces, or, roused by a feeling of their insignificance, seek to emulate her noble example. Age is now creeping on apace, yet her love for her profession still burns with its youthful fervor, and the desire to benefit her race still warms her heart. Sweet must be the rest which age brings to her latter days, as with joy in duty done she looks back to the grateful hearts which beat with loving adoration, and lips that called her blessed.

My task is done. All I have said may seem only the inspiration of youthful enthusiasm, soon to be lost when the routine of practice takes the place of scientific ardor. Yet if the subject receives serious consideration, and the suggestions here given be carried out, good will follow, and I shall rejoice that my task has not been in vain.

